# Surgical Management of a Right Coronary Artery Fistula After Failed Transcatheter Closure: A Case Report

**DOI:** 10.7759/cureus.80361

**Published:** 2025-03-10

**Authors:** Alassal Ahmed, Lamia A Alkodami, Mustafa A AlMuhaya, Shadha A Al-Zubaidi, Nizam Uddin, Mansour Almutairi

**Affiliations:** 1 Cardiac Surgery, Madina Cardiac Center, Madina, SAU; 2 Internal Medicine, Sulaiman Alrajhi University, Medina, SAU; 3 Pediatric Cardiology, King Abdulaziz Medical City, Madina, SAU

**Keywords:** congenital anomalies of coronary arteries, congenital coronary anomaly, congenital coronary artery anomaly, coronary arterial fistula, coronary cameral fistula, right coronary artery (rca)

## Abstract

A coronary artery fistula (CAF) is an abnormal communication between a coronary artery and a great vessel or cardiac chamber. CAFs are very rare and are mostly of congenital origin, but they can also be acquired. Patients are usually asymptomatic since the majority of CAFs are small in size; hence, most cases are usually discovered incidentally during routine angiographic investigations. Moderate or large CAFs can be symptomatic, causing angina pectoris due to coronary steal phenomenon, and can carry a high risk of serious complications such as myocardial infarction, heart failure, or even sudden cardiac death. Consequently, prompt management with the appropriate choice of intervention - surgical versus transcatheter closure - is essential to prevent such complications.

We present a case of a 52-year-old male patient with a right coronary artery-coronary sinus fistula managed with surgical closure.

## Introduction

The pathological findings of congenital coronary artery fistulas (CAFs) were first identified by Krause in the postmortem of a male patient in 1865 [1]. A CAF is defined as an anomalous communication between a coronary artery and a heart chamber (coronary-cameral fistula) or a great vessel (coronary-vascular fistula) such as the coronary sinus or pulmonary artery [2]. CAFs can be congenital or acquired, and they may occur either in isolation or in association with other congenital heart diseases [2].

Patients with small CAFs are often asymptomatic, resulting in most diagnoses being made incidentally during angiographic evaluations or upon investigation for a heart murmur [3]. However, moderate or large CAFs can be symptomatic, and patients frequently experience angina or ischemic chest pain as a result of the coronary steal phenomenon, where blood is redirected away from some areas of the myocardium, especially when there is increased demand for blood flow.

The prevalence of complications related to CAFs is 11% in patients younger than 20 years and 35% in those over 20 years of age. Some complications associated with CAFs include myocardial infarction, heart failure due to fluid overload, bacterial endocarditis secondary to turbulent flow, and, rarely, spontaneous rupture of the dilated coronary artery due to weakening of the vessel wall. Given these potential adverse consequences, early diagnosis and prompt treatment are significant [2-3].

In this paper, we describe a rare case of an adult male patient with a right coronary artery-coronary sinus fistula managed surgically after failed transcatheter closure of the fistula. We also discuss the radiological, angiographic, and surgical findings along with the management plan.

## Case presentation

A 52-year-old male patient, with a medical history of diabetes and hypertension, was referred to our institution with the diagnosis of a right coronary artery fistula, which was identified due to symptoms, for further management.

At presentation, he was asymptomatic but reported a history of recurrent, severe, retrosternal pain, that started three years ago. It was intermittently worsening in frequency and intensity in the six months leading up to admission. Moreover, each episode lasted for a few seconds and was exacerbated at night. It was associated with orthopnea, exercise-induced shortness of breath, and fatigue. He denied any previous cardiac diseases and systemic review analysis ruled out any other comorbidities.

During the initial workup, physical examination showed the following: pulse rate 75 beats/minute, respiratory rate 15 breaths/minute, blood pressure 140/80 mmHg, oxygen saturation 95%, and temperature 37.2 °C. A cardiovascular examination revealed no murmurs. Pulmonary, abdominal, and peripheral vascular examinations were unremarkable.

**Figure 1:**
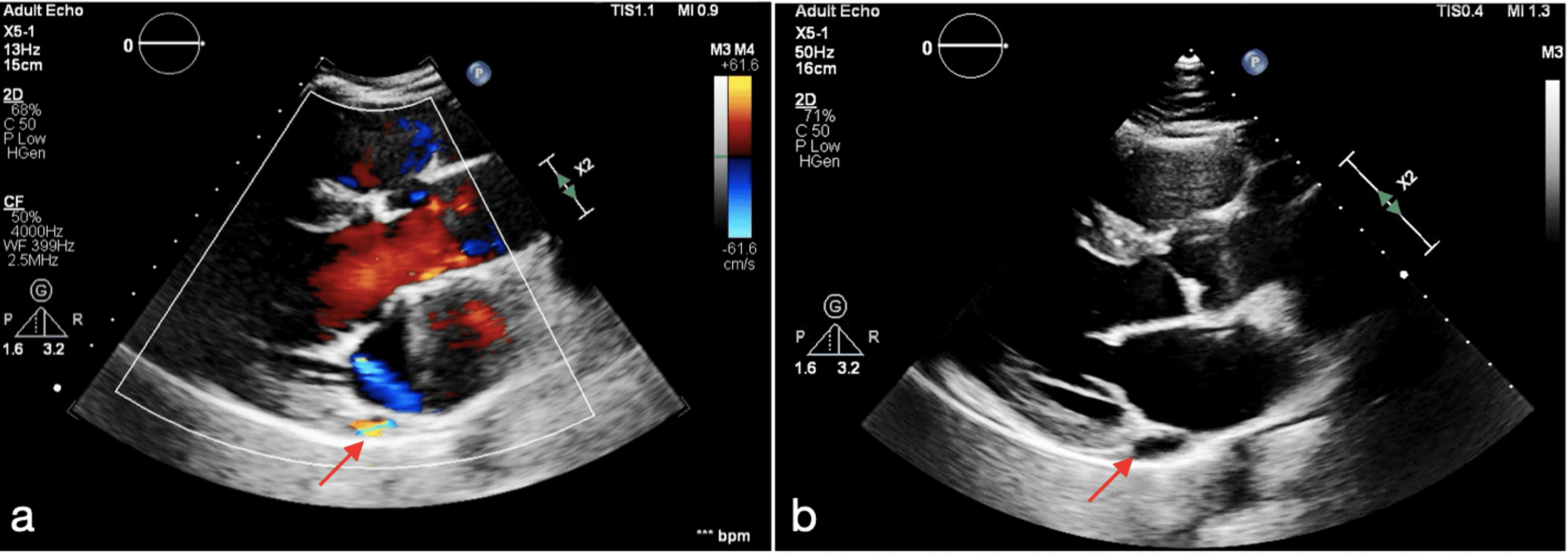
Abnormal blood flow through a fistula from the right coronary artery to the coronary sinus (red arrow in image A) and a prominent coronary sinus with abnormal blood flow (red arrow in image B)

Repeated laboratory tests showed normal values and cardiac markers were within normal range. ECG and chest X-ray showed no gross abnormalities. Subsequently, detailed echocardiography (Figure 1) showed abnormal blood flow through a fistula connecting the right coronary artery to a dilated coronary sinus, probably causing coronary steal phenomenon and ischemic symptoms.

It also revealed a mildly enlarged right atrium, caused by volume overload from the fistula, which explains the patient’s orthopnea and exertional dyspnea. The aorta, pulmonary artery, and pericardium were normal.

**Figure 2:**
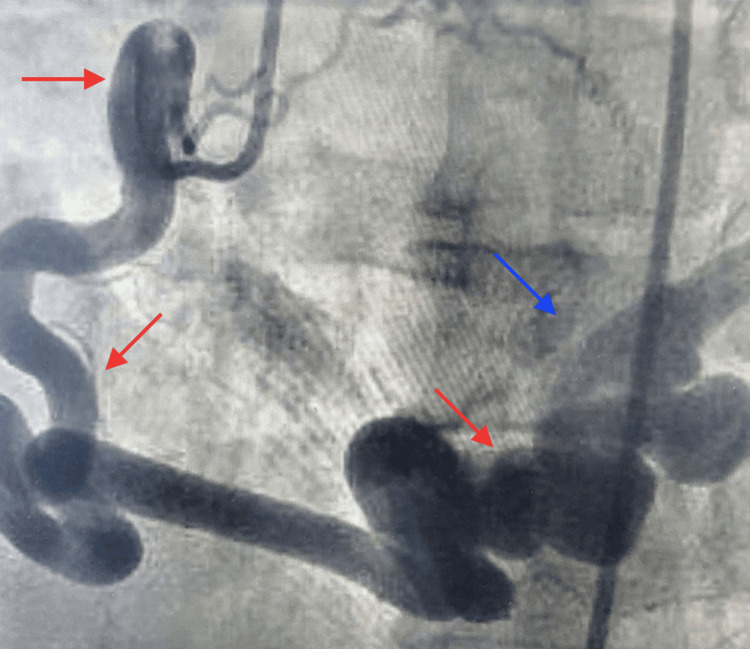
Tortuous RCA with multiple hugely dilated loops (red arrows) and an abnormal blood flow to the coronary sinus (blue arrow) RCA: right coronary artery

**Figure 3:**
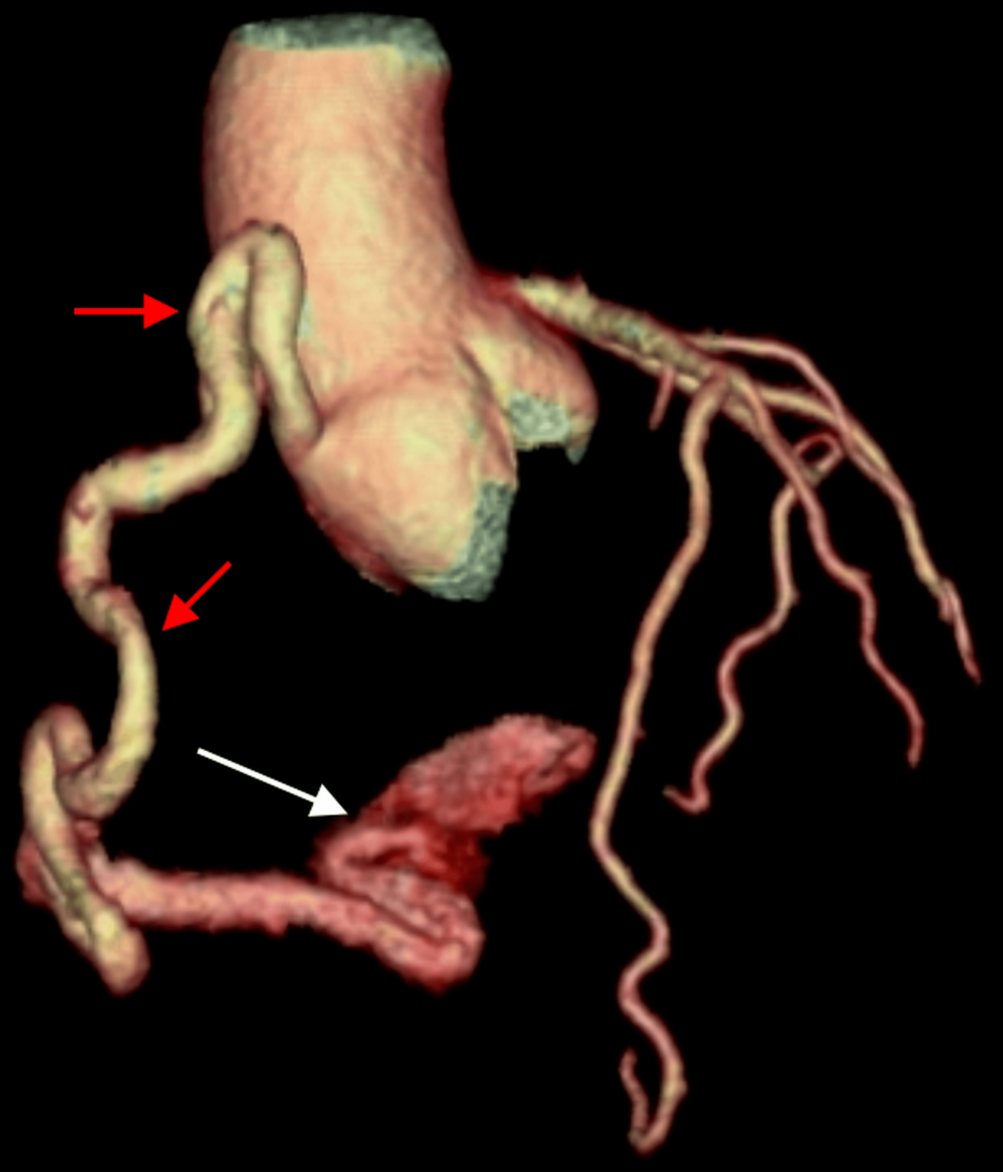
Tortuous, dilated right coronary artery (red arrows) and a large-sized fistula opening into the coronary sinus (white arrow) RCA: right coronary artery

Coronary angiography (Figure 2) showed a tortuous right coronary artery (RCA) with multiple hugely dilated loops and an abnormal blood flow to the coronary sinus. Cardiac CT angiography (Figure 3) also demonstrated a tortuous, dilated RCA and a large-sized fistula (>2 times the largest diameter of the coronary vessel not feeding the coronary fistula) opening into the coronary sinus.

The patient's symptoms, explained by the imaging results, and the size of the CAF highlight the urgent need for intervention to relieve these symptoms and decrease the risk of serious complications, such as myocardial infarction.

Initially, an attempt was made to manage the fistula through transcatheter closure (TCC). The interventional team first used the right common femoral artery with an antegrade approach trying to navigate the tortuous loops of the RCA and reach the fistula's main opening. However, after multiple unsuccessful attempts with arterial access due to the anatomical challenges presented by the RCA's tortuosity, they switched to a retrograde approach via the right femoral vein to the right atrium and coronary sinus. This method also proved unsuccessful because it wasn't possible to cross from the CS to the fistulous tract. Consequently, the procedure was aborted and the plan was to discuss the patient's condition for possible surgical management. Subsequently, it was agreed in the multidisciplinary heart team meeting (HTM) to proceed with surgical ligation of the fistulous tract.

After a routine workup for cardiac surgery, the patient was taken to the operating room and prepped according to standard protocol. A transesophageal echocardiography (TEE) probe was inserted preoperatively to demonstrate the abnormal blood flow from the RCA through the fistula to the coronary sinus (CS) and right atrium and to monitor any change in this flow after surgical ligation. Subsequently, median sternotomy was performed and the pericardium was opened and fixed with stay sutures.

The RCA morphology (Figure 3) was as follows: the RCA had abnormally dilated, massive loops with a diameter exceeding 1 cm originating from an enlarged right coronary sinus in the aortic root. The most proximal loop was coursing superiorly toward the ascending aorta to a point above the level of the sinotubular junction, then turning inferiorly to make an inverted U shape and continuing in the AV groove. The middle portion was severely tortuous with a convoluted appearance (explaining why it was difficult to reach by catheter). The distal segment of the RCA had a short straight segment that became again tortuous and significantly convoluted at the level of the crux cordis before continuing as the posterolateral ventricular artery.

After dissection from the myocardium, the fistulous tract was identified and snared temporarily along its course and a few small RCA branches, which could be possibly communicating with the tract of the fistula, were clipped. Simultaneously, the abnormal flow to the coronary sinus and the right atrium disappeared on the TEE. Watchful observation for abnormalities in cardiac contractility on TEE and ischemic changes or arrhythmias on ECG was conducted for more than 30 minutes. Since the cardiac function and the ECG remained normal, definitive surgical ligation of the fistulous tract was carried out. Further observation post-ligation was performed and final hemostasis was checked before closure of the median sternotomy incision.

After surgery, the patient was shifted to the Cardiac Surgical Intensive Care Unit (CSICU) in stable condition. Extubation was withheld for 24 hours to assess the cardiac function by echocardiography and to observe the trends of the cardiac enzymes as well as to monitor for any complications or pathological ECG changes. The cardiac enzymes and ECG showed favorable findings, so the patient was extubated on the first post-operative day. There were no major concerns during the rest of his CSICU stay, so he was transferred to the adult cardiac surgical ward on the second postoperative day for further recovery and preparation for discharge. In the subsequent days, his pre-discharge ECHO remained satisfactory, and he was discharged free of symptoms with a plan for follow-up at the Outpatient Department at 1, 6, and 12 months. The patient was discharged on long-term antiplatelet therapy (Aspirin) and other routine post-cardiac surgery medications.

**Figure 4:**
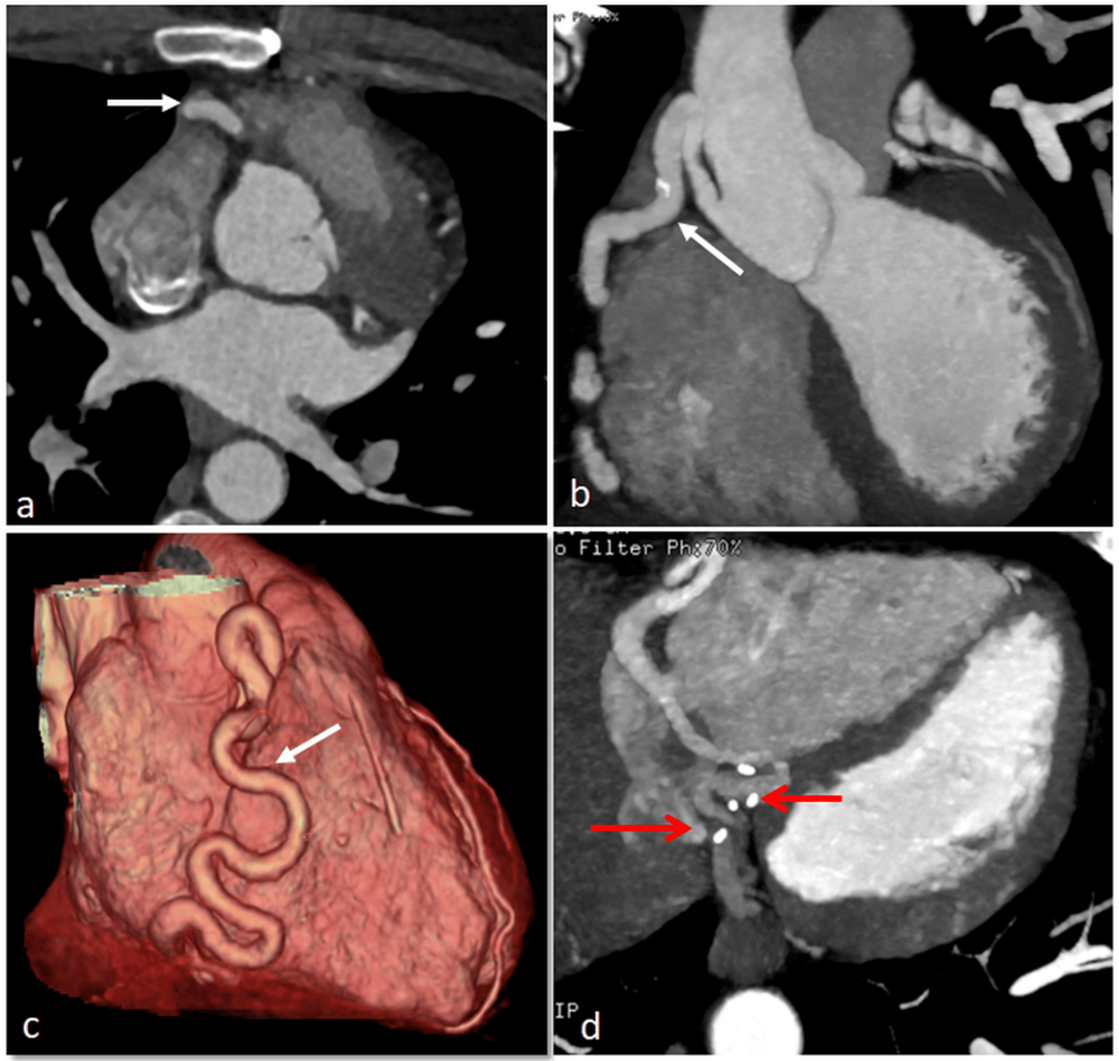
Reduction in the right coronary artery size (white arrows), with surgical clips ligating the small RCA branches that are potentially feeding the ligated fistula (not shown) to prevent recurrence (red arrows) RCA: right coronary artery

During the follow-up period, the patient reported complete resolution of symptoms, and all postoperative investigations yielded satisfactory findings, confirming the success of the surgical intervention. The echocardiogram at six months showed good cardiac function and the complete absence of abnormal blood flow within the coronary sinus and right atrium. Cardiac CT angiography (Figure 4) showed a remarkable reduction in the RCA size, and serial ECG during the follow-up period did not reveal any ischemic changes. The same findings were also observed 12 months postoperatively.

## Discussion

Normally, patients with congenital moderate-to-large CAFs manifest with symptoms of angina, cardiomyopathy, or heart failure early on in life. This leads to the early discovery of CAFs. However, most small-sized CAFs tend to be asymptomatic and close spontaneously, making them undetectable. On some occasions, small CAFs persist and become symptomatic only during adulthood, most likely due to a gradual increase in the size of the fistula over several years, becoming hemodynamically significant and causing symptoms. This progressive enlargement of the fistula can lead to coronary steal phenomenon, which in turn causes the coronary vessel to dilate as an adaptive compensatory response and can progress to aneurysm formation. Physical examination of CAFs usually reveals no abnormalities except for an occasionally documented continuous murmur on the lower precordium in some patients [2].

Echocardiography can usually detect most CAFs; however, it is not sufficient on its own to confirm the diagnosis preoperatively due to limited visualization of the fistula's course and drainage sites, particularly in cases with complex anatomy, as it cannot isolate the adjacent structures from the fistula. Conventional coronary angiography can also be used, and it is the gold standard investigation for CAFs. However, less invasive investigations, such as cardiac CT angiography (CCTA) and multidetector CT (MDCT) can also help in diagnosing CAFs accurately. MDCT has the benefits of being non-invasive and successfully illustrating details regarding the CAF’s morphology (size and location) and its relation relative to the neighboring heart structures separately in two or three dimensions. We recommend the use of several cardiovascular imaging modalities for the investigation of complicated cases, including echocardiography, CCTA or MDCT, and invasive coronary angiography [3].

The first report of a successful surgical closure of a CAF was reported by Bjork et al. in 1947 [4]. Since then, surgical closure of a CAF remained the main management option. In 1983, Reidy et al. performed the first transcatheter closure of a CAF, adding another approach for treating CAFs [5]. Manoly et al. have also presented a hybrid approach that combines catheter isolation of fistulas to locate drainage sites followed by cardiac surgery [6]. This method allowed for precise preoperative evaluation of the CAF morphology, making it easier for the surgeon to trace the course of the fistula and enhancing the precision and outcome of the procedure. However, this hybrid technique has not been widely implemented, and this could be due to the limited availability of resources and cost considerations since it would require both a cardiac surgeon and an interventional cardiologist to perform the procedure.

There is still controversy regarding choosing the best management strategy for CAFs. Recommendations for choosing a specific strategy depend on multiple factors, such as the size and anatomy of the fistula, symptomatology, and presence of other cardiac lesions. Most authors consider that symptomatic patients should receive invasive management [2,7], but there is still debate regarding the treatment of asymptomatic patients.

The American Heart Association (AHA)/American College of Cardiology (ACC) 2008 Guidelines for Adults with Congenital Heart Disease (ACHD) categorized their recommendations according to the size and symptomatology of the CAFs. They indicated a class I recommendation for transcatheter or surgical occlusion of large CAFs [8]. They instructed that the fistula should be occluded after delineation of its course and after assessment of the potential of either approach to fully obliterate it, irrespective of the presence of symptoms (level of evidence: C). They further emphasized that only those interventional cardiologists and surgeons with training and expertise in congenital heart diseases should perform the operations (level of evidence: C).

Moreover, the AHA/ACC also indicated a class I recommendation for closing small-to-moderate CAFs via either transcatheter embolization or surgical intervention, if associated with other cardiac conditions such as myocardial ischemia, arrhythmias, unexplained ventricular systolic/diastolic dysfunction or enlargement, or endarteritis (level of evidence: C).

In addition, they recommend that small, asymptomatic CAFs discovered incidentally should not undergo closure (Class III, level of evidence: C). However, serial clinical evaluation with echocardiography every three to five years should be done to exclude any changes in the pathology that might change the management plan. These changes include progression of the size of the fistulous tract due to age-related physiological or pathological changes, development of symptoms, or occurrence of complications such as arrhythmias or enlargement of the heart chambers (Class IIa, level of evidence: C).

Furthermore, the 2011 AHA Guidelines on Indications for Cardiac Catheterization and Intervention in Pediatric Cardiac Disease have put forward more restrictive recommendations. They indicated a class IIa recommendation to utilize transcatheter occlusion as the management for patients with moderate or large CAFs without clinical symptoms (level of evidence: C) [9]. Other indications for closure also include: a pulmonary to systemic blood flow ratio (Qp/Qs ratio) >1.5-1.7, worsening pulmonary hypertension, congestive heart failure, signs of ischemia or volume overload in the right ventricle on echocardiography, a history of infective endocarditis, and aneurysm formation [10].

The data on the comparisons of the efficacy and safety between surgical closure (SC) and transcatheter closure (TCC) of CAFs is still limited. In a retrospective study conducted by Wang et al., they compared the success rate and the complications during hospitalization and at follow-up of both SC and TCC in pediatric patients [11]. In total, 121 pediatric patients with congenital CAFs were divided into TCC (63 patients; 52.1%) and SC (58 patients; 47.9%) groups according to indications. They found that, compared to the TCC group, the incidence of intra-operative blood loss (19.7 ± 12.8 mL vs 109.7 ± 66.6 mL; p < 0.001) and the proportion of patients requiring blood transfusion were higher (3.2% vs 58.6%; p < 0.001) in the SC group. These patients also required a longer duration of hospital and ICU stay (7.8 ± 4.0 days vs 14.2 ± 4.4 days; p < 0.001). On the contrary, major complications, such as myocardial ischemia (10.2% vs 0.0%, p=0.028), residual shunts (16.9% vs 3.6%, p=0.045), and new-onset moderate-to-severe valve regurgitation (11.9% vs 0.0%, p=0.013), were higher in the TCC group during follow-up. In addition to having a lower risk of major complications, SC had a higher rate of successful closure in pediatric patients.

Yihang Li et al. also reported that the procedural success rate for SC was higher (93.3%) as compared to 85.2% for TCC in 42 consecutive adult patients, but the results were not statistically significant (P = 0.639) [12]. Patients who underwent TC had a significantly shorter postoperative in-hospital length of stay (2.11 vs. 7.73 days, P<0.001), and there was no difference in the incidence of recanalization of the fistula (7.4% vs. 6.7%, P = 1) and myocardial infarction (0% vs. 0%). Additionally, two patients in the TC group developed cerebral infarction due to the discontinuation of anticoagulants, and* *seven patients were found to have thrombotic occlusion of the fistulous tract with a patent parent coronary artery.

Although the percutaneous technique offers the advantages of being less invasive, requiring a shorter time for recovery [12-13], causing less morbidity, and therefore being more suited for patients with a high perioperative risk profile, it is not always applicable because of its strict eligibility criteria. These criteria are the absence of a concomitant cardiac disorder requiring surgical intervention and favorable anatomy, which includes non-tortuous vessels, an accessible fistula that can be reached with the closure device, and a fistula with distal narrowing to avoid embolism to the drainage site. Additionally, large branch vessels that can be accidentally embolized, complicated communications with neighboring structures, and multiple drainage sites are not preferred [14-16]. Possible procedural complications of TCC include transient ischemic changes or atrial arrhythmia, device migration and embolization, fistula dissection, and myocardial infarction [7].

In our case, the transcatheter technique was chosen initially based on the recommendations of experts in the previous institution, which later referred the case to us. Since this approach failed, surgical closure was considered as an alternative treatment. Surgical management offers the superior option if the following indications are present: distal fistulas, high-flow fistulas, tortuous arteries, prominent aneurysms, a wide drainage site, and the presence of large, complex vascular branches that can be accidentally embolized if the percutaneous approach is used [10].

Although most of the case series report an excellent procedural success rate, there is limited information about the prognosis of patients with a CAF treated surgically. Said et al. reported short-term and long-term complications in 46 adult patients who underwent surgical closure of congenital CAFs [1]. In the short term, early mortality occurred in 1 patient (2%) within 5 days postoperatively due to a massive myocardial infarction, and 5 patients (11%) experienced postoperative MI, with 2 of these patients dying from this incident. Moreover, early reoperation was required in two patients (4%) due to postoperative bleeding. In the long term, 11 patients (24%) died during the follow-up period, with 1 death being attributed to acute MI and 10 to non-cardiac causes. Late reoperation was needed in three patients (6.5%) for pre-existing valvular disorders. Additionally, one patient (2%) developed a recurrent coronary fistula during the follow-up while residual fistulas were found in three patients (6%), which did not require re-intervention as they were not hemodynamically significant. The long-term survival was 93%, 74%, and 68% at 1, 5, and 15 years, respectively. There is no difference in survival between those who were younger than 60 years old and those who were older (P = .13). The overall survival of congenital CAF patients was significantly lower than that of a matched Minnesotan white population (P = .031).

## Conclusions

Currently, there are no standardized guidelines for choosing between surgical or percutaneous closure of CAFs, leaving the option to the discretion of the operating surgeon. A study by Al-Hijji et al. attempted to establish an algorithm for the management of CAFs based on the size of the fistula, presence of other surgical indications, and anticipated benefits and risks of both procedures. However, this study, along with other anecdotal studies, are unofficial recommendations that are not implemented in the management of all CAFs. For this reason, professional societies should take the lead in developing guidelines that lay down the indications for either approach to be used.

This case and many other similar cases in the literature emphasize the need for establishing such criteria to avoid inappropriate selection of treatment choices for fistulas and to improve patient outcomes. By adopting these guidelines, we can significantly reduce complication rates, enhance procedural success rates, and promote longer-term survival. Careful selection of the closure method optimizes patient health by improving the effectiveness of the intervention.
